# Assisted mechanical ventilation: the future is now!

**DOI:** 10.1186/s12871-015-0092-y

**Published:** 2015-07-29

**Authors:** Robert M Kacmarek, Massimiliano Pirrone, Lorenzo Berra

**Affiliations:** Massachusetts General Hospital and Harvard Medical School, Boston, Massachusetts USA

## Abstract

Assisted ventilation is a highly complex process that requires an intimate interaction between the ventilator and the patient. The complexity of this form of ventilation is frequently underappreciated by the bedside clinician. In assisted mechanical ventilation, regardless of the specific mode, the ventilator’s gas delivery pattern and the patient’s breathing pattern must match near perfectly or asynchrony between the patient and the ventilator occurs. Asynchrony can be categorized into four general types: flow asynchrony; trigger asynchrony; cycle asynchrony; and mode asynchrony. In an article recently published in BMC Anesthesiology, Hodane et al. have demonstrated reduced asynchrony during assisted ventilation with Neurally Adjusted Ventilatory Assist (NAVA) as compared to pressure support ventilation (PSV). These findings add to the growing volume of data indicating that modes of ventilation that provide proportional assistance to ventilation – e.g., NAVA and Proportional Assist Ventilation (PAV) – markedly reduce asynchrony. As it becomes more accepted that the respiratory center of the patient in most circumstances is the most appropriate determinant of ventilatory pattern and as the negative outcome effects of patient-ventilator asynchrony become ever more recognized, we can expect NAVA and PAV to become the preferred modes of assisted ventilation!

## Background

Global approaches to mechanical ventilation (MV) are usually categorized as controlled or assisted. During controlled MV the patient has no role is the process of gas delivery. As a result of pharmacological control of neural/muscular drive, each breath is programmed and delivered without active patient interaction. For this reason, controlled mechanical ventilation is generally considered a relatively simple process. Assisted ventilation, on the other hand, is a highly complex process that requires an intimate interaction between the ventilator and the patient. The complexity of this form of ventilation is frequently underappreciated by the bedside clinician. In assisted mechanical ventilation, regardless of the specific mode, the ventilator’s gas delivery pattern and the patient’s breathing pattern must match near perfectly or asynchrony between the patient and the ventilator occurs.

## Main text

Asynchrony in conventional modes of MV is a problem because such modes control one or more of the following ventilation variables: pressure, flow, volume or time. The greater the number of these variables controlled by the ventilator, the greater the likelihood that asynchrony will be present. In Volume Assist/Control (VA/C) mode, the clinician sets tidal volume, peak flow, flow pattern and inspiratory time either directly or as a result of the interaction between variables. In either case, the only variable that can possibly vary on a breath-to-breath basis is airway pressure. Thus, the patient’s respiratory center must adjust to a precise tidal volume delivered with a precise flow pattern and flow rate in a precisely set inspiratory time. This process is difficult for the respiratory center and often results in patient-ventilator asynchrony during VA/C ventilation. Pressure A/C (PA/C) is less imposing since in this mode only airway pressure and inspiratory time are controlled by the ventilator. The patient has the ability to control flow rate and pattern and, as a consequence, tidal volume. PSV is the least controlling of the classic ventilatory modes since the only variable the clinician sets is airway pressure. With all of these modes of ventilation, patients are required to follow the lead of the ventilator and to adjust their respiratory center output to match the way the clinician sets the ventilator, or the result is asynchrony.

Asynchrony can be categorized into four general types: flow asynchrony; trigger asynchrony; cycle asynchrony; and mode asynchrony. With flow asynchrony, the gas delivery pattern from the ventilator does not match the inspiratory pattern of the patient [1, 2]. Thus, when the patient’s inspiratory flow exceeds the ventilator’s delivered flow, patient effort and work of breathing increase stimulating an increased respiratory rate. This type of asynchrony is much more common during volume ventilation than pressure ventilation [1, 2].

Trigger asynchrony can be of several forms: trigger delay; missed triggering; auto-triggering; or double triggering. Trigger delay can occur in any mode as a result of poor setting of the trigger sensitivity [3]. Missed triggering is primarily a result of auto-PEEP [4]. Auto-triggering, a result of inappropriate setting of the trigger sensitivity, system leaks, or a hyperdynamic cardiac output (as sometimes observed in post cardiac surgical patients), can also occur in any mode [5, 6]. Double triggering occurs when the tidal volume delivered by the ventilator is less than that demanded by the respiratory center or when the inspiratory time set on the ventilator is shorter than the neuro-inspiratory time. Double triggering is most common with volume ventilation [7, 8].

Cycle asynchrony occurs when the inspiratory time of the patient and the ventilator do not match and is referred to as short cycling or long cycling [9, 10]. This form of asynchrony is most common during pressure ventilation but may also cause double triggering in volume ventilation [9, 10].

Mode asynchrony occurs when the selected mode results in a significant level of asynchrony.

Recently, increased attention has been given to the presence of asynchrony in mechanically ventilated patients. Data would indicate that all patients ventilated in assisted ventilation have periods of asynchrony and that in many the level of asynchrony can be excessive [11]. A number of groups have shown that an asynchrony index greater than or equal to 10 % (total number of asynchronous breaths/total triggered and untriggered breathes ×100) is associated with an increased length of mechanical ventilation [12, 13], intensive care unit and hospital length of stay [12, 13], and mortality [11]. The potential impact of asynchrony on patient outcome has been generally underappreciated by the practicing clinician.

In an article recently published in BMC Anesthesiology, Hodane et al. [14] have demonstrated reduced asynchrony during assisted ventilation with Neurally Adjusted Ventilatory Assist (NAVA) as compared to PSV. They studied 30 patients with stable respiratory failure who were randomly assigned to 23 hrs of PSV and 23 hrs of NAVA. Patient waveforms were collected for the complete 23 hr period, and 5 min of waveforms every 4 hrs were analyzed manually for the presence of asynchrony. They identified a greater number of asynchronies per hour in PSV and a greater asynchrony index in PSV. In NAVA, the percentage of missed triggers and auto-triggering was lower than in PSV, but double triggering was higher in NAVA. The higher level of double triggering may be a result of the inspiratory termination criteria during NAVA. As with PSV, NAVA terminates inspiration when delivered flow decreases to a percentage of peak flow. Adjustment of this setting to a lower percentage would prolong the NAVA breath and possibly reduce the double triggering.

These findings add to the increasing volume of data indicating that modes of ventilation that provide proportional assistance to ventilation – e.g., NAVA and Proportional Assist Ventilation (PAV) – markedly reduce asynchrony [15–18]. The reason is these modes of ventilation “do NOT control” the patient’s ventilatory pattern. In both modes the patient is allowed to select whatever pattern the respiratory center considers appropriate. Neither pressure, flow, volume nor time is set; all that is set is the proportion of effort provided by the ventilator to supplement the patient’s effort. As a result, these modes FOLLOW the lead of the patient but again “do NOT force” a ventilatory pattern. Thus, it can be expected that NAVA and PAV should result in decreased asynchrony compared to all classic modes of ventilatory support [19].

## Conclusion

The major conceptual difficulty with NAVA and PAV is the “inability of the clinician to control” the patient’s ventilatory pattern. We in medicine prefer to control the application of mechanical ventilation; these modes do not allow for this. As it becomes more accepted that the respiratory center of the patient in most circumstances is the most appropriate determinant of ventilatory pattern and as the negative outcome effects of patient-ventilator asynchrony become ever more recognized, we can expect NAVA and PAV to become the preferred modes of assisted ventilation.

## Abbreviations

MV: Mechanical Ventilation
VA/C: Volume Assist/Control
PA/C: Pressure A/C
PSV: Pressure Support Ventilation
NAVA: Neurally Adjusted Ventilatory Assist
PAV: Proportional Assist Ventilation
